# Elastic Restraint Effect of Concrete Circular Columns with Ultrahigh-Performance Concrete Jackets: An Analytical and Experimental Study

**DOI:** 10.3390/ma14123278

**Published:** 2021-06-14

**Authors:** Mujahed Alsomiri, Xiaofang Jiang, Zhao Liu

**Affiliations:** 1 School of Civil Engineering, Southeast University, Nanjing 210018, China; mujahed.seu@yahoo.com (M.A.); jiangxiaofang2021@163.com (X.J.); 2 Key Laboratory of Concrete and Prestressed Concrete Structures of Ministry of Education, School of Civil Engineering, Southeast University, Nanjing 210018, China

**Keywords:** concrete circular columns, strengthening, retrofitting, deterioration, UHPC, elastic restraint, jacketing

## Abstract

Concrete circular columns are among the most common vertical load-bearing members in structural engineering. Because of the change of service loads or environmental factors, the strengthening of deteriorated members is often demanded to restore and maintain their performance. In view of the limitations of the traditional strengthening methods and the superior mechanical properties of the new material, ultra-high-performance concrete (UHPC), this study analyzed the stress–strain state of concrete circular columns confined by UHPC jackets under axial compression in the elastic stage. Since elastic analysis is the basis for the service limit state design, the elastic stress solution was derived through the theory of elasticity, and experimental verification of the effectiveness of the UHPC jackets in circular concrete columns was performed. Theoretical bases and references for practical strengthening works are provided.

## 1. Introduction

In bridge and building structures, reinforced concrete columns are among the most common vertical load-bearing components. When columns in bridges or building structures experience an increase in service loads, damage caused by earthquakes or deterioration due to exposure to physical and chemical attacks in harsh environments, strengthening and rehabilitation are often demanded to restore and enhance their performance [1,2,3]. In the new era, the rehabilitation and strengthening of structures have been endorsed by many researchers as more sustainable solutions than demolition and reconstruction of the whole structure.

During the last few decades, extensive research has been devoted to developing methods for the rehabilitation of concrete columns [4]. Of note, conventional jacketing with reinforced concrete has been found to be an effective technique in enhancing the load-carrying capacity and ductility of members [5,6]. Additional steel reinforcement layers are supplied in this method, and thick concrete coating is usually used [7]. This method, however, results in enlarging the cross-section of the member, thereby increasing its self-weight and sectional properties, which might not be favourable because of aesthetic reasons or dynamic design considerations.

Fibre-reinforced polymer (FRP) wrapping has also been adopted in the external confinement of columns [8,9,10,11] and has been shown to be efficient in improving the stiffness, strength, and durability of the members, and it offers long-term economic benefits [12]. However, specific preparations such as processing epoxy resin are needed to guarantee firm interface bonding between FRP sheets and concrete surfaces, which may be costly if large retrofitting quantities are requested [13,14]. Besides, the durability at the interfaces cannot be ensured, especially in high chloride environments [15,16].

Steel plate jacketing has enriched the retrofitting techniques. The abundant availability of steel makes it an attractive option for strengthening existing deteriorated columns [17]. In this technique, external pressure was suggested to attach the steel jackets to the column’s surface to ensure a good interface connection, and double-layered steel jackets and corrugated steel plates were also proposed to improve the ductility, energy dissipation, and seismic-resistance capacity [18,19]. Nevertheless, steel is generally susceptible to corrosion, and its durability is a serious problem when considered for the retrofitting of structures located in aggressive environments [20,21].

In addition to the retrofitting technologies mentioned above, other techniques based on novel materials have also been employed, incorporating cementitious fibre-reinforced concrete [22,23,24], textile cement-based matrices [25,26,27,28], and engineered cementitious composites (ECC) [28,29,30]. Moreover, high-performance concrete (HPC) has garnered interest as a potential solution to overcome shortcomings and limitations in durability, interface bonding, and capacity [31,32,33]. In recent times, ultra-high-performance concrete (UHPC), the most innovative concrete product, has been widely promoted for a variety of applications in the construction industry, i.e., bearing components, deck pavements, bridge joints, blast-resistant structures, tunnel lining segments, and the retrofitting of existing structures [34,35,36,37]. UHPC seems to be a promising retrofitting technology because of its superior mechanical properties, high compressive strength, and sustained post-cracking tensile strength provided by fibres [38,39,40]. Additionally, UHPC exhibits a noticeable toughness and energy dissipation capacity [41,42,43] and has good compatibility with normal concrete [44], along with its favourable characteristics in terms of durability, sustainability, and recyclability [45,46,47,48].

**Figure 1:**
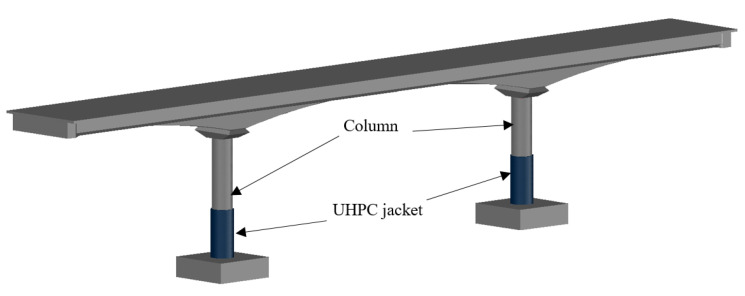
Application of UHPC jacketing.

Recently, several studies have been dedicated to probing the applicability and significance of UHPC in the jacketing of bridge columns [42,43,49,50], as illustrated in Figure 1. Ichikawa et al. [41] examined the response of three reduced-scale columns retrofitted with UHPC jackets at the plastic hinge regions under axial and bilateral cyclic loading. It was observed that columns with UHPC jackets demonstrated better performance and energy dissipation and reduced the plastic hinge damage. Similarly, Tong et al. [42,43] studied the seismic response of as-built large-scale bridge piers with different configurations of UHPC jackets enclosing the plastic hinge zones under cyclic loading. The passive confinement provided by UHPC jackets was significant, and higher seismic-resistant capacities and ductility were achieved. 

Farzad et al. [50,51] investigated the structural performance and durability of columns with UHPC jackets subjected to axil and cyclic lateral loading. In their studies, numerical and analytical models were proposed for predicting the service life of the retrofitted columns, and the experimental outcomes indicated that UHPC shells efficiently enhanced the lateral strength, deformation, and energy dissipation of the damaged elements. Besides, it was remarked that the UHPC shell converts abrupt cover spalling to a gradual slow mechanism as steel fibres help sustain the integrity of the member and reduce the propagation of cracks.

The compression behaviour of columns with UHPC jackets has also been explored. Xie, Fu, and Yan [52] analytically and experimentally investigated 18 square and circular UHPC-jacketed columns with variable jacket thicknesses. Their study revealed that UHPC jacketing does not considerably increase the strength of square sections compared to its prominent influence on circular sections. In another study, Dadvar, Mostofinejad, and Bahmani [53] examined the axial behaviour of 14 circular reinforced concrete columns with UHPC jackets taking into account the influence of the jacket’s thickness, the fibre type, and the different sets of groovings intended to achieve monolithic compression behaviour in the entire specimen. The results showed that jackets with steel fibres and longitudinal groovings had a higher contribution to the load-carrying capacity and energy absorption than those with macro-fibres and horizontal groovings. 

To the best of the authors’ knowledge, most of the research conducted through the last few years on the influence of UHPC jacketing on columns was primarily focused on enhancing or restoring the strength under seismic loading. Undoubtedly, strength is a priority to ensure the safety of the structure, and the corresponding ultimate limit state criteria should be satisfied. However, the service limit states of columns with UHPC jackets have not been fully addressed in the previous works, and the elastic confinement imposed by UHPC jackets has not been quantified and is usually overlooked. The elastic restraint effect could come into play when the service loading conditions change, e.g., increased heavy traffic loads on bridges whose columns are designed to maintain an elastic state. Therefore, the influence of UHPC jackets on the elastic enhancement of columns still requires further research.

The main objective of the present study was to evaluate and quantify the elastic restraint effect of circular concrete columns strengthened with UHPC jackets under axial loading. An analytical solution describing the stresses and strains of the jacketed columns was developed through the theory of elasticity, and experimental tests on small-scale circular columns were performed for validation. The effectiveness of the UHPC jacketing as a strengthening method for concrete circular columns is discussed, and a theoretical basis and reference for practical strengthening works are provided.

## 2. Analytical Solution for the Elastic Restraint Effect of Concrete Circular Columns with UHPC Jackets

**Figure 2:**
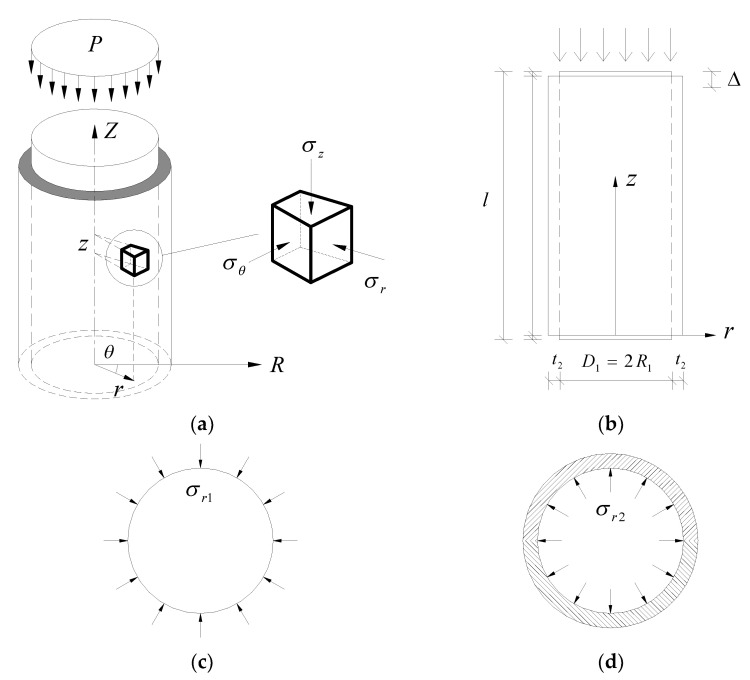
Schematic diagram of a concrete cylinder with UHPC jacket under axial compression: (**a**) 3D view; (**b**) side view; (**c**) radial stress of concrete cylinder; (**d**) radial stress on UHPC jacket.

The concrete circular column is prototyped by a concrete cylinder, presented in the schematic illustration in Figure 2. The normal concrete stub cylinder is wrapped by a UHPC jacket (Figure 2a), and the UHPC jacket is slightly shorter than the stub cylinder at the top and bottom in order not to be directly compressed under axial pressure *P*. The height of the confined concrete cylinder is *l*, the radius of the cylinder is *R*_1_, the elastic modulus and Poisson’s ratio of the cylinder are *E*_1_ and *μ*_1_, respectively. The thickness of the UHPC jacket layer is *t*_2_, the elastic modulus and Poisson’s ratio of the UHPC are *E*_2_ and *μ*_2_, respectively. The self-weights of the stub cylinder and the jacket are neglected. 

The stresses and strains in the concrete cylinder are triggered by the occurrence of the vertical displacement Δ, and the confinement effect of the UHPC jacket is assumed to be activated at the instant the load is applied, i.e., a passive confinement effect.

According to the theory of elasticity [54], the equilibrium differential equations of the space axisymmetric problem in the polar coordinate system are:

(1) $$\frac{\partial \sigma_{r}}{\partial r}+\frac{\partial \tau_{zr}}{\partial z}+\frac{\sigma_{r}-\sigma_{\theta }}{r}=0$$

(2) $$\frac{\partial \sigma_{z}}{\partial z}+\frac{\partial \tau_{zr}}{\partial r}+\frac{\tau_{zr}}{r}=0$$

When the concrete cylinder is subjected to axial uniform compression, the radial shear stress *τ_zr_* = 0. Given that, Equations (1) and (2) can be rewritten as:

(3) $$\frac{\partial \sigma_{r1}}{\partial r}+\frac{\sigma_{r1}-\sigma_{\theta 1}}{r}=0$$

(4) $$\frac{\partial \sigma_{r1}}{\partial z}=0$$

The strains of the concrete cylinder in polar coordinates are [54]:

(5) $$\varepsilon_{r1}=\frac{1}{E_{1}}[\sigma_{r1}-\mu_{1}(\sigma_{\theta 1}+\sigma_{z1})]$$

(6) $$\varepsilon_{\theta 1}=\frac{1}{E_{1}}[\sigma_{\theta 1}-\mu_{1}(\sigma_{r1}+\sigma_{z1})]$$

(7) $$\varepsilon_{z1}=\frac{1}{E_{1}}[\sigma_{z1}-\mu_{1}(\sigma_{\theta 1}+\sigma_{r1})]$$

Additionally, the strains as functions of displacements in polar coordinates are:

(8) $$\varepsilon_{r1}=\frac{du_{r1}}{dr}$$

(9) $$\varepsilon_{r1}=\frac{du_{r1}}{dr}$$

(10) $$\varepsilon_{z1}=\frac{\partial u_{z1}}{\partial z}=\frac{\Delta }{l}$$

Taking the vertical, radial, and tangential stresses, *σ_r_*_1_, *σ_θ_*_1_ and *σ_z_*_1_, respectively, in Equations (5)–(7) as unknowns and solving by substitution or subtraction, the following expressions are obtained:

(11) $$\sigma_{r1}=\frac{E_{1}}{\left(1+\mu_{1}\right)\left(1-2\mu_{1}\right)}\left[\left(1-\mu_{1}\right)\varepsilon_{r1}+\mu_{1}\varepsilon_{\theta 1}+\mu_{1}\varepsilon_{z1}\right]$$

(12) $$\sigma_{\theta 1}=\frac{E_{1}}{\left(1+\mu_{1}\right)\left(1-2\mu_{1}\right)}\left[\left(1-\mu_{1}\right)\varepsilon_{\theta 1}+\mu_{1}\varepsilon_{r1}+\mu_{1}\varepsilon_{z1}\right]$$

(13) $$\sigma_{\theta 1}=\frac{E_{1}}{\left(1+\mu_{1}\right)\left(1-2\mu_{1}\right)}\left[\left(1-\mu_{1}\right)\varepsilon_{\theta 1}+\mu_{1}\varepsilon_{r1}+\mu_{1}\varepsilon_{z1}\right]$$

By substituting Equations (8)–(10) into Equations (11)–(13), *σ_r_*_1_, *σ_θ_*_1_ and *σ_z_*_1_ can be related to *u_r_*_1_. Then, substituting the results into Equations (3) and (4), the following expression is derived:

(14) $$r^{2}\frac{d^{2}u_{r1}}{dr^{2}}+r\frac{du_{r1}}{dr}-u_{r1}=0$$

Equation (14) is a second-order differential equation, and its general solution takes the form:

(15) $$u_{r1}=A_{1}r+\frac{B_{1}}{r}$$

Because the radial displacement of the cylinder on the axis of symmetry is zero, the constant *B*_1_ = 0. Therefore, the radial displacement of the concrete cylinder under axial uniform compression is linearly related to *r*:

(16) $$u_{r1}=A_{1}r$$

By substituting Equation (16) into Equations (8) and (9), the radial and tangential strains are obtained:

(17) $$\varepsilon_{r1}=\varepsilon_{\theta 1}=A_{1}$$

When the vertical displacement of the cylinder occurs, the vertical compressive strain is simply given as:

(18) $$\varepsilon_{z1}=\frac{\Delta }{l}$$

Substituting Equations (17) and (18) into Equations (11)–(13), *σ_r_*_1_, *σ_θ_*_1_ and *σ_z_*_1_ can be obtained as:

(19) $$\sigma_{r1}=\sigma_{\theta 1}=\frac{E_{1}}{\left(1+\mu_{1}\right)\left(1-2\mu_{1}\right)}(A_{1}+\mu_{1}\frac{\Delta }{l})$$

(20) $$\sigma_{z1}=\frac{E_{1}}{\left(1+\mu_{1}\right)\left(1-2\mu_{1}\right)}[2\mu_{1}A_{1}+(1-\mu_{1})\frac{\Delta }{l}]$$

The strains of the UHPC jacket in polar coordinates are:

(21) $$\varepsilon_{r2}=\frac{1}{E_{1}}[\sigma_{r2}-\mu_{1}(\sigma_{\theta 2}+\sigma_{z2})]$$

(22) $$\varepsilon_{\theta 2}=\frac{1}{E_{1}}[\sigma_{\theta 2}-\mu_{1}(\sigma_{r2}+\sigma_{z2})]$$

(23) $$\varepsilon_{z2}=\frac{1}{E_{1}}[\sigma_{z2}-\mu_{1}(\sigma_{\theta 2}+\sigma_{r2})]$$

**Figure 3:**
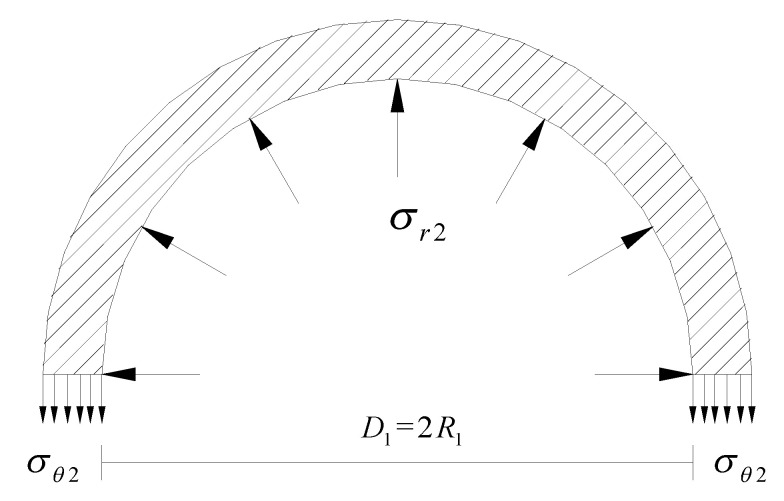
Equilibrium condition of the UHPC jacket.

Assuming that the UHPC jacket is a thin-walled member in which the hoop stress *σ_θ_*_2_ can be simplified as uniformly distributed along the jacket thickness, the equilibrium condition of the UHPC jacket is established (see Figure 3). 

From the equilibrium condition and by substituting Equation (22), the radial stress at the interface can be expressed as:

(24) $$\sigma_{r2}=-\frac{\sigma_{\theta 2}t_{2}}{R_{1}}=-\frac{\left(\varepsilon_{\theta 2}E_{2}+\mu_{2}\sigma_{r2}\right)t_{2}}{R_{1}}$$

Rearranging Equation (24) gives:

(25) $$\sigma_{r2}=-\frac{\varepsilon_{\theta 2}E_{2}t_{2}}{R_{1}+t_{2}\mu_{2}}$$

On the contact surface between the UHPC jacket and the concrete cylinder, the radial contact stress condition is:

(26) $$\sigma_{r1}(R_{1})=\sigma_{r2}(R_{1})$$

By substituting Equations (19) and (25) into Equation (26), the following is obtained:

(27) $$\frac{E_{1}}{\left(1+\mu_{1}\right)\left(1-2\mu_{1}\right)}(A_{1}+\mu_{1}\frac{\Delta }{l})=-\frac{\varepsilon_{\theta 2}E_{2}t_{2}}{R_{1}+\mu_{2}t_{2}}$$

Based on the strain compatibility condition on the contact interface of the UHPC jacket and concrete cylinder, Equation (17) can be rewritten as:

(28) $$\varepsilon_{\theta 1}(R_{1})=\varepsilon_{\theta 2}(R_{1})=A_{1}$$

Replacing *ε_θ_*_2_ in Equation (27) with the undetermined coefficient *A*_1_ according to Equation (28), and then solving for *A*_1_, gives:

(29) $$A_{1}=-\frac{\mu_{1}}{1+\frac{\left(1+\mu_{1}\right)\left(1-2\mu_{1}\right)t_{2}E_{2}}{\left(R_{1}+\mu_{2}t_{2}\right)E_{1}}}\frac{\Delta }{l}$$

By substituting Equation (29) into Equation (17), the analytical solution of the radial and tangential strains of the cylinder under compression can be expressed as:

(30) $$\varepsilon_{r1}=\varepsilon_{\theta 1}=-\frac{\mu_{1}(\lambda +\mu_{2})}{\lambda +\mu_{2}+\left(1+\mu_{1}\right)\left(1-2\mu_{1}\right)n_{E}}\frac{\Delta }{l}$$

where:

(31) $$\lambda =R_{1}/t_{2}$$

(32) $$n_{E}=E_{2}/E_{1}$$

Moreover, substituting the value of *A*_1_ from Equation (18) into Equations (19) and (20), the stresses in the cylinder under compression are:

(33) $$\sigma_{r1}=\sigma_{\theta 1}=\frac{\mu_{1}n_{E}}{\lambda +\mu_{2}+\left(1+\mu_{1}\right)\left(1-2\mu_{1}\right)n_{E}}\frac{\Delta }{l}E_{1}$$

(34) $$\sigma_{z1}=\frac{(\lambda +\mu_{2})+\left(1-\mu_{1}\right)n_{E}}{\lambda +\mu_{2}+\left(1+\mu_{1}\right)\left(1-2\mu_{1}\right)n_{E}}\frac{\Delta }{l}E_{1}$$

Adhering to the assumption that the hoop stress and strain do not vary along the thickness of the thin-walled UHPC jacket and substituting Equations (33) and (34) into Equations (24) and (26), the stresses in the UHPC jacket can be obtained as follows:

(35) $$\sigma_{r2}=\sigma_{r1}=\frac{\mu_{1}n_{E}}{\lambda +\mu_{2}+\left(1+\mu_{1}\right)\left(1-2\mu_{1}\right)n_{E}}\frac{\Delta }{l}E_{1}$$

(36) $$\sigma_{\theta 2}=-\lambda \sigma_{r2}=-\frac{\lambda \mu_{1}n_{E}}{\lambda +\mu_{2}+\left(1+\mu_{1}\right)\left(1-2\mu_{1}\right)n_{E}}\frac{\Delta }{l}E_{1}$$

The tangential strain of the UHPC jacket can be obtained by substituting Equation (22) into Equation (17), which yields:

(37) $$\varepsilon_{\theta 2}=-\frac{\mu_{1}(\lambda +\mu_{2})}{\lambda +\mu_{2}+\left(1+\mu_{1}\right)\left(1-2\mu_{1}\right)n_{E}}\frac{\Delta }{l}$$

The radial strain of the UHPC jacket is also obtained by substituting Equations (35) and (36) into Equation (21), expressed by:

(38) $$\varepsilon_{r2}=\frac{\mu_{1}(1+\lambda \mu_{2})}{\lambda +\mu_{2}+\left(1+\mu_{1}\right)\left(1-2\mu_{1}\right)n_{E}}\frac{\Delta }{l}$$

## 3. Experimental Program

### 3.1. Specimens Fabrication

Three small-scale column specimens were prepared, one of which was plain normal concrete (RC), and the other two were jacketed specimens (J1, J2). The total height of the specimen was 600 mm, the diameter of the stub column was 250 mm, and the UHPC jacket thickness was 25 mm. The height of the UHPC jacket was 580 mm and was 10 mm shorter at the top and bottom to guarantee that the specimen was not directly compressed and that only the confinement effect was imposed by the jacket.

**Figure 4:**
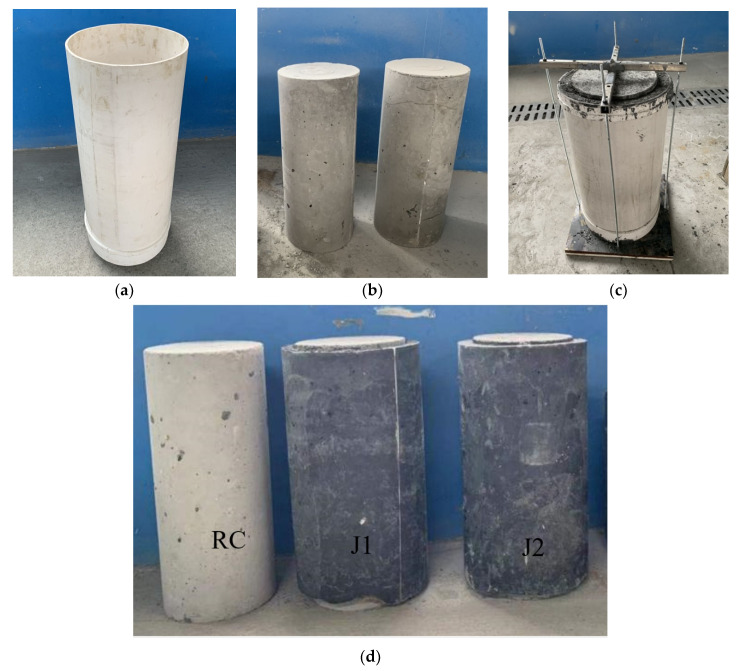
Preparation of test specimens: (**a**) PVC mould; (**b**) plain concrete cylinders; (**c**) UHPC jacket casting; (**d**) final forms of specimens.

The fabrication of the specimens, illustrated in Figure 4, was implemented in the following order. PVC moulds for the concrete stub columns and the UHPC jackets were prepared according to the required geometry. The plain normal concrete mixture was cast into the PVC moulds. Simple water-cover curing under room temperature was adopted, and the specimens were left for 28 days to obtain their design strength. Then, a commercial UHPC mixture, detailed in Table 1, with 2% by volume randomly distributed steel fibres, was prepared and cast into the gap between the external mould and the stub column. 

The type of steel fibres used in this study was straight, and they had a 0.2 mm diameter and were 20 mm long, with a tensile strength of 2600 MPa, an elastic modulus of 205 GPa, and a density of 7840 kg/m^3^. At the time of casting the UHPC, slight vibration was used on the specimens to assure good compactness. The curing conditions of the UHPC jackets were identical to those of the normal concrete stub columns. After reaching the design strength, the moulds were removed, and the specimens’ final forms were checked to have met the requirements. Rebar reinforcement, either longitudinal or transverse, was not used in this study, following the assumption that the reinforcement is almost inactive in the elastic stage prior to cracking. 

**Figure 5:**
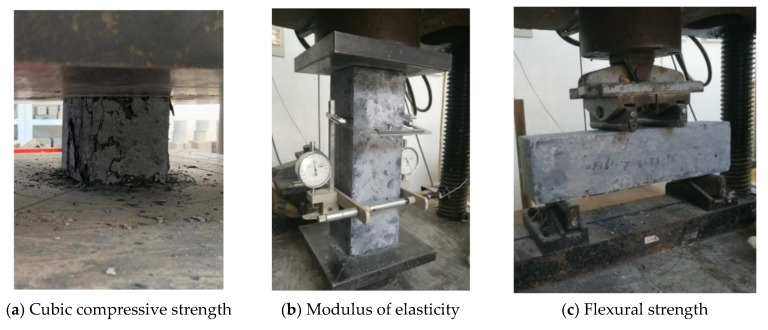
Tests of UHPC mechanical properties.

During the casting of the primary specimens, samples for material properties characterization were prepared and tested. Only a compression test was performed for normal concrete, and the corresponding elastic modulus and tensile strength were determined according to the GB/T standard [55]. The material properties of the normal concrete are listed in Table 2. On the other hand, three sets of tests were performed to find the compressive strength, the elastic modulus, and the flexural strength of UHPC, shown in Figure 5. The obtained results are given in Table 3. 

### 3.2. Specimens Loading

**Figure 6:**
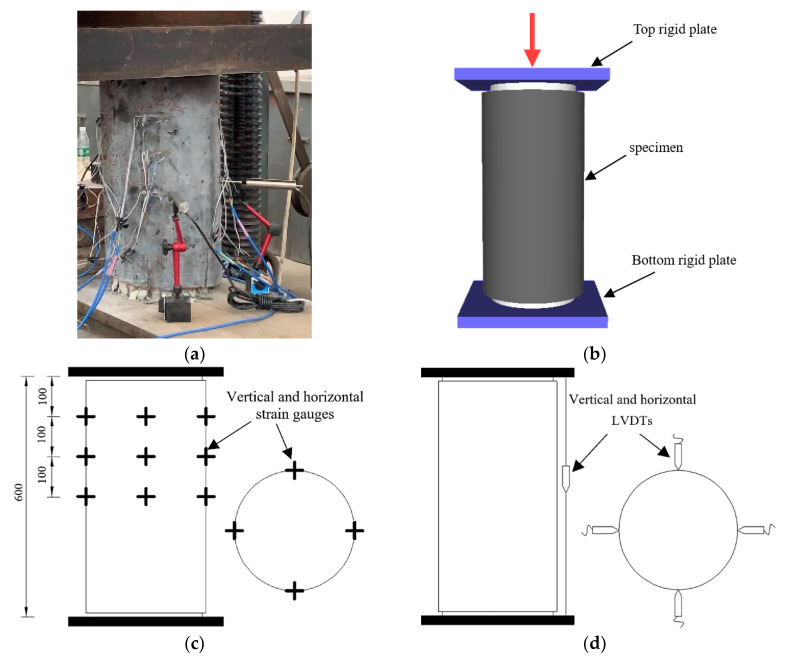
Test set-up: (**a**) test machine; (**b**) loading scheme of specimen; (**c**) strain gauges arrangement (unites are in mm); (**d**) displacement meters.

A 5000 kN pressure testing machine was used to apply monotonic axial loading on the three specimens (see Figure 6a). The axial loading scheme on the specimen is shown in Figure 6b. In each specimen, three layers of vertical and horizontal strain gauges were installed around the surface of the upper half of the specimen (Figure 6c) to comprehensively measure the strains and capture the response during the loading process. In addition, a total of 8 linear variable differential transformers (LVDTs), vertically and horizontally arranged, were attached to the sides of the specimen to measure the vertical and transverse displacements, illustrated in Figure 6d.

A data acquisition system (DAS) was used to collect the stresses, displacements, and strains data throughout the experiment, and cracking was monitored from the moment the load was applied until the collapse of the specimen. A load-controlled approach was adopted in this experimental program. The incremental loading step was 50 kN for the RC specimen and 100 kN for the J1 and J2 specimens. The accuracy and reliability of this test approach have been established in previous studies [52,53] 

### 3.3. Test Results and Failure Modes

A summary of the test results is given in Table 4. The ultimate bearing capacity of the RC specimen was 1850 kN, whereas the J1 and J2 specimens reached values of 2800 and 2650 kN, respectively, which was about 50% higher than the RC. The coefficient of variation of the ultimate load for J1 and J2 specimens was 3.89%, and the corresponding standard deviation was 106 kN, indicating good accuracy. Likewise, the compressive strength of the jacketed samples was also increased by an average of 55%. Besides, the samples with the UHPC jackets, J1 and J2, exhibited excellent deformation capacity and achieved values of an ultimate displacement 90% larger than that of the RC. The reason for the higher deformation capacity (ductility) can be attributed to the high tensile strength of UHPC and the sustained post-cracking performance provided by the fibres. Since the confinement effect significantly depends on the material capability to withstand in-plane tension, as in hoop stresses, the tensile performance of UHPC is a key characteristic in the application of jacketing. This remark is evident in the obtained results. The increase in the load-bearing capacity and the improvement in the ductility provided by the UHPC jackets observed herein agrees with previous studies [43,50,51,52,53]. 

**Figure 7:**
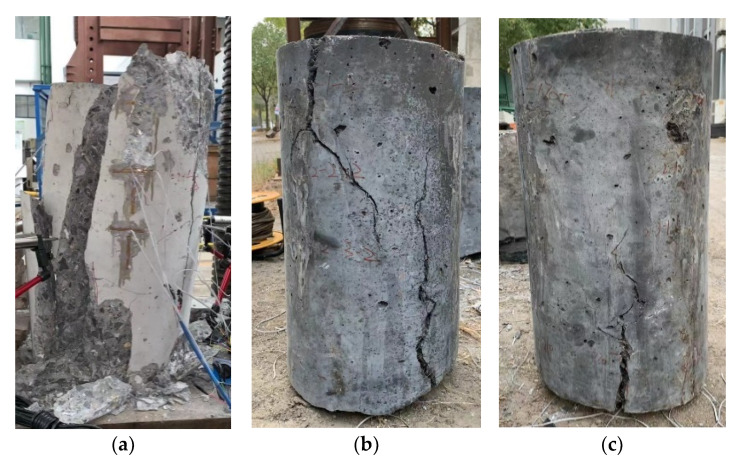
Failure state of specimens: (**a**) RC; (**b**) J1; (**c**) J2.

The ultimate failure modes of the three specimens are shown in Figure 7. The cracks on the unconfined RC specimen took the form of splitting cracks that completely separated the specimen into two parts. The failure mechanism of the RC specimen was brittle and explosive. It should be mentioned that this failure scenario would have improved if spiral rebar reinforcements were used. On the other hand, the generated cracks in J1 and J2 specimens were parallel to the direction of the loading with limited extension at the upper and lower parts of the specimens with slight inclinations at the middle. No cracks were observed to extend through the full height of the specimens. In contrast to the RC specimen, the J1 and J2 specimens maintained their original forms, and the cracking mechanism was ductile and gradual. 

**Figure 8:**
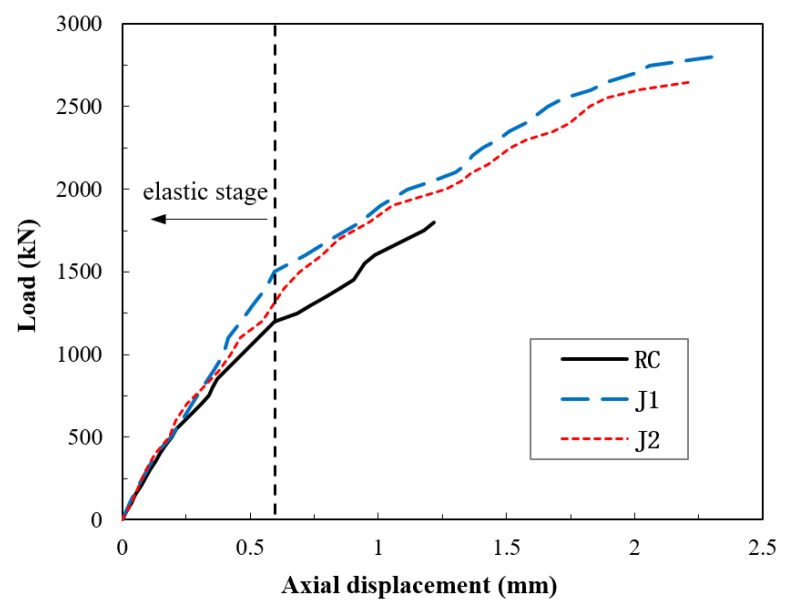
Test results for load–axial displacement curves.

**Figure 9:**
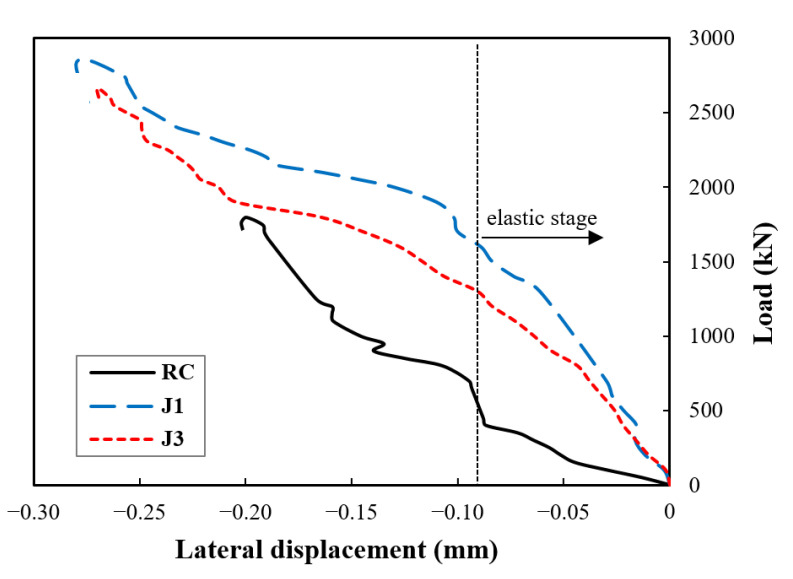
Test results for load-axial displacement curves.

The test results for the load versus axial displacement curves are shown in Figure 8. It can be seen that the specimens demonstrated a linear elastic behaviour until the applied load reached about 55% of the ultimate load; this observation conforms with the findings of a previous study [52]. The load versus the lateral displacement of the specimens are also presented in Figure 9. The ultimate lateral displacement of J1 and J2 was 35% higher than that of RC. The confinement effect at the elastic stage was manifested more in the lateral displacement. The J1 and J2 specimens displayed a slower displacement rate than the RC due to the counteract of the jackets on restraining the lateral deformations. After reaching the cracking points, at which the nonlinear phase started, the influence of the confinement became more significant as the UHPC composite transitioned into the hardening phase (multiple cracking), which can be clearly seen in Figure 7b,c. Therefore, the jackets not only increased the ultimate displacements but also slowed down the displacement rate. The post-peak behaviour did not occur in the specimens because a load-controlled approach was used rather than a displacement or strain-controlled loading. Accordingly, the peak load was the collapse load, and the peak displacement was equal to the ultimate displacement. 

**Figure 10:**
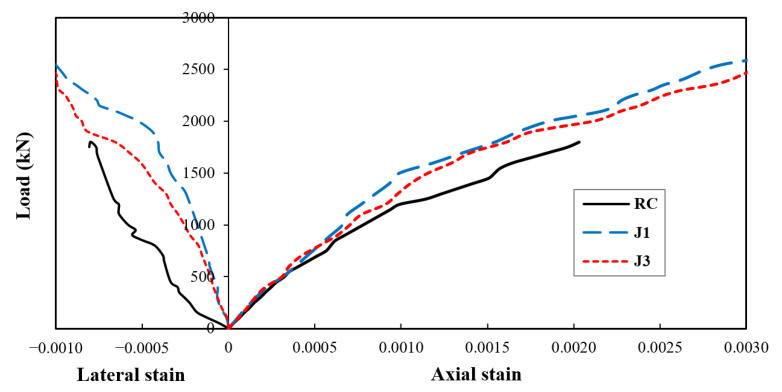
Test results for strains.

The axial and lateral strains results obtained from the test are shown in Figure 10. The maximum axial strain achieved by the RC specimen was 0.002, whereas the J1 and J2 specimens demonstrated a much better response, reaching a value of 0.0038, about 90% higher than that of the RC specimen. Similarly, the maximum lateral strain of the RC was 0.00075, whereas the J1 and J1 specimens surpassed a value up to 0.0012. The boost in the lateral strain provided by the jackets was 60%. 

**Figure 11:**
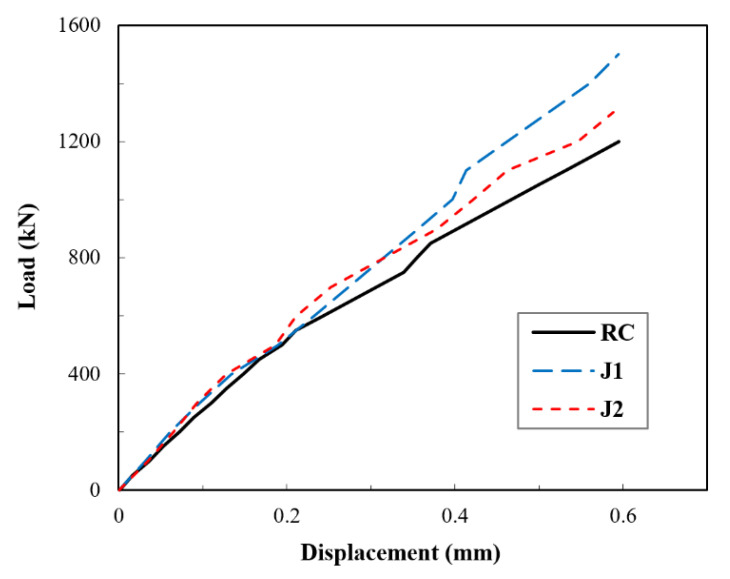
Test results for load–displacement curves in elastic stage.

In compliance with the emphasis of this study, which is the elastic restraint effect, the load–axial displacement curves in the elastic stage were extracted and are shown in Figure 11. The elastic response considered here incorporated the elastic and the microcracking phases. From Figure 11, it can be observed that even in the elastic stage, jackets in the J1 and J2 specimens were effective. This effect was usually overlooked in the previous studies, and only the nonlinear enhancement was discussed. Indeed, the significance of the jackets is more manifested in the nonlinear stage, as previously mentioned; yet, the effect seems to be non-negligible when looking closely at the linear response. The effectiveness of UHPC jacketing in the elastic stage was ascribed to the excellent compatibility and adhesion between UHPC and normal concrete, as both materials are cement-based. This feature plays an essential role in the activation of the jacket at early displacement, roughly upon the application of the load. Treatments such as vibration used during casting help accomplish good compactness within the UHPC jacket and the good adhesion at the contact interface of the UHPC and stub column, indicating the importance of casting treatments.

**Figure 12:**
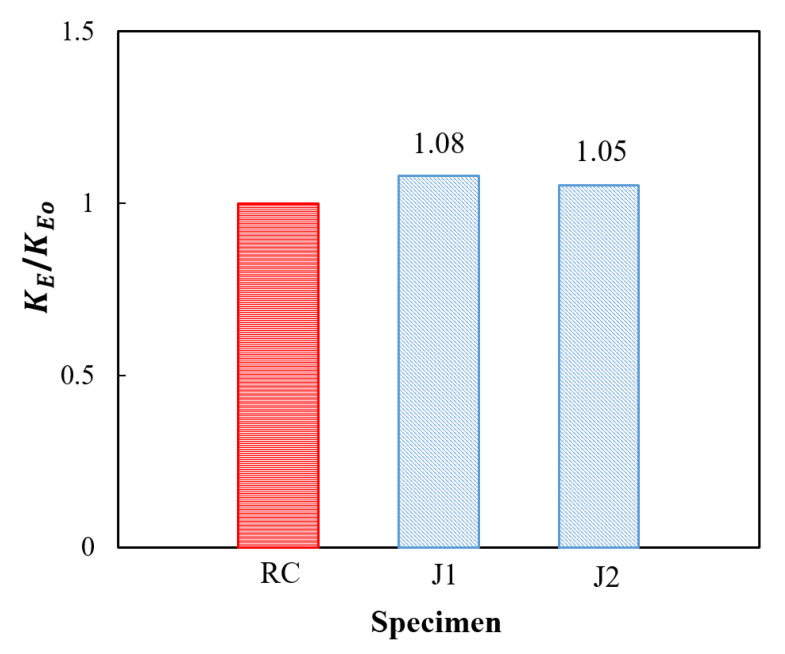
Elastic stiffness.

Figure 12 shows a comparison of the secant elastic stiffness *K_E_*. The elastic stiffness imposed by the UHPC jackets was increased by 8% and 5% for specimens J1 and J2, respectively. This percentage gives a good recommendation to use UHPC jacketing to restore the elastic capacity that may be degraded because of the reduction in the cross-section area when the outer side of the column is abraded in severe environments. In addition, the elastic stiffness is needed to be increased without much enlargement of the cross-section.

## 4. Verification of the Elastic Restraint Effect of Concrete Circular Columns with UHPC Jackets

### 4.1. Vertical Load–Displacement Curve and Constraint Effect Analysis

**Figure 13:**
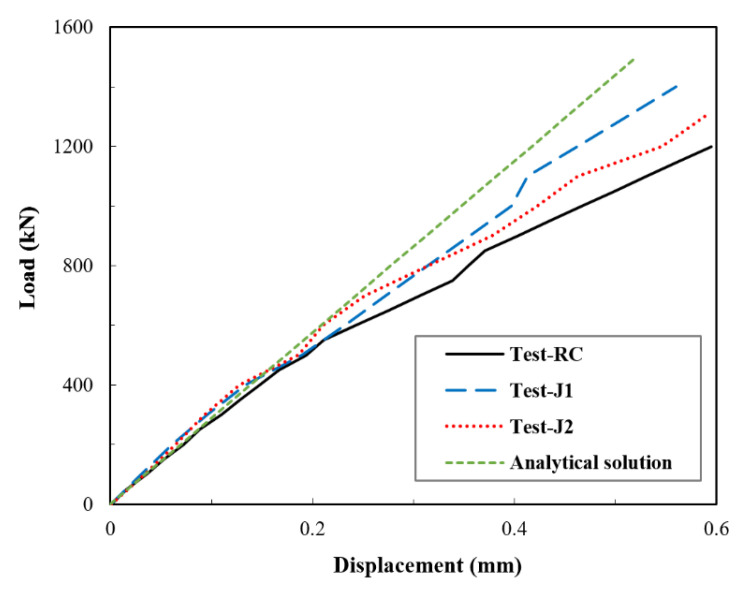
Comparison between test results and the analytical solution for load–axial displacement curves.

The load–axial displacement curves as per the analytical solution given in Equation (22) and the test data were compared, as shown in Figure 13. The results showed that the analytical solution gave a slightly overestimated response compared to the test results. This overestimation may be attributed to the formation of unnoticeable concealed microcracks that occurred in the normal concrete stub columns in the test, but which were not taken into account in the analytical solution. The formation of microcracks, due to the weak interfacial bond between the aggregate and the paste matrix, has been reported to start immediately upon loading [57]. Such an effect is frequently ignored but detectable in load–displacement curves, and this case is an obvious instance. The difference between the analytical solution and the test results remained almost constant, and the response remained fairly linear until the load reached about 55% of the ultimate load. After this point, microcracking increased noticeably, and the response took a nonlinear form, as shown in Figure 8. Nevertheless, the load–displacement values obtained from the analytical solution were in an acceptable agreement with the tests and gave a good estimation.

In Equation (34), a coefficient *K* is defined as:

(39) $$K=\frac{(\lambda +\mu_{2})+\left(1-\mu_{1}\right)n_{E}}{\lambda +\mu_{2}+\left(1+\mu_{1}\right)\left(1-2\mu_{1}\right)n_{E}}$$

Accordingly, Equation (34) can be rewritten as:

(40) $$\sigma_{z1}=K\frac{\Delta_{1}}{l}E_{1}$$

Here, *K* is called the restraint effect coefficient of the UHPC jacket, which represents the increase in the elastic vertical compressive resistance of the concrete circular column with the UHPC jacket when a vertical displacement Δ_1_ occurs. In other words, the coefficient *K* reflects the improvement of the vertical deformation resistance of the column supplied by the UHPC jacket. For a regular circular concrete column without a UHPC jacket, the vertical stress is given by:

(41) $$\sigma_{z0}=\frac{\Delta_{0}}{l}E_{1}$$

When a specific axial load is applied to the column, the value of the induced stress is equal in both types of specimen *σ*_*z*0_ = *σ*_*z*1_. Then, the relationship between the vertical displacement of the concrete column with UHPC jacket Δ_1_ and that without UHPC jacket Δ_0_ can be expressed by:

(42) $$K=\frac{\Delta_{1}}{\Delta_{0}}$$

**Figure 14:**
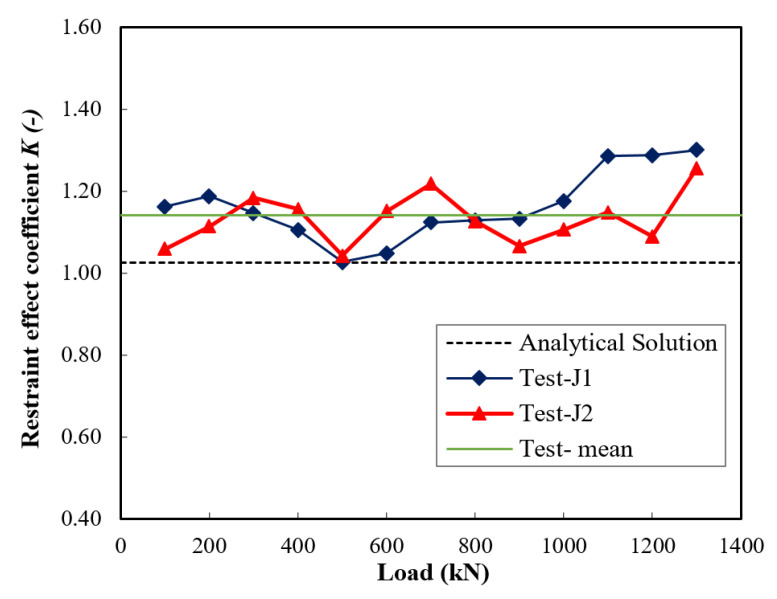
Comparison of the restraint effect coefficient *K*.

The results of the restraint effect coefficient *K* obtained from the test and the analytical solution throughout loading until the end of the elastic stage are shown in Figure 14. The range of *K* values obtained from the tests was between 1.04 and 1.3, and the average was 1.14. When the restraint coefficient was calculated using the analytical solution, an improvement in the vertical deformation resistance of about 6% was achieved. The reason for this comparably lower value was the overestimated linear response given by the analytical solution, which gave a stiffer response for both types of columns with and without jackets. 

### 4.2. Circumferential (Hoop) Strain Analysis of UHPC Jackets

**Figure 15:**
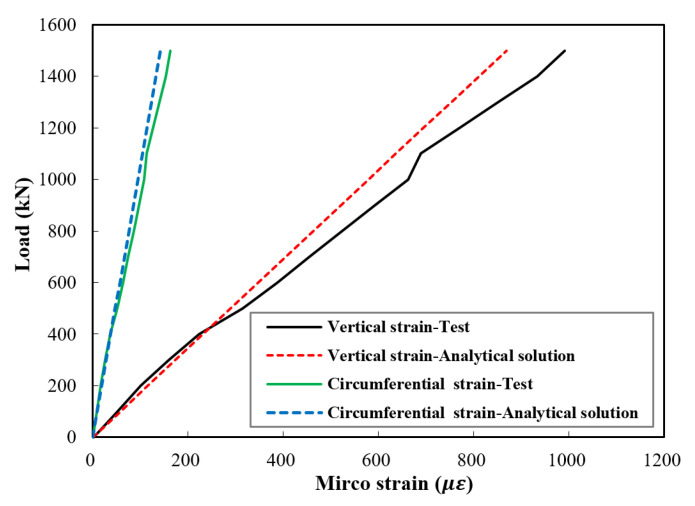
Comparison between test results and analytical solution of the vertical strain and the circumferential strain of UHPC jacket of specimen J1.

**Figure 16:**
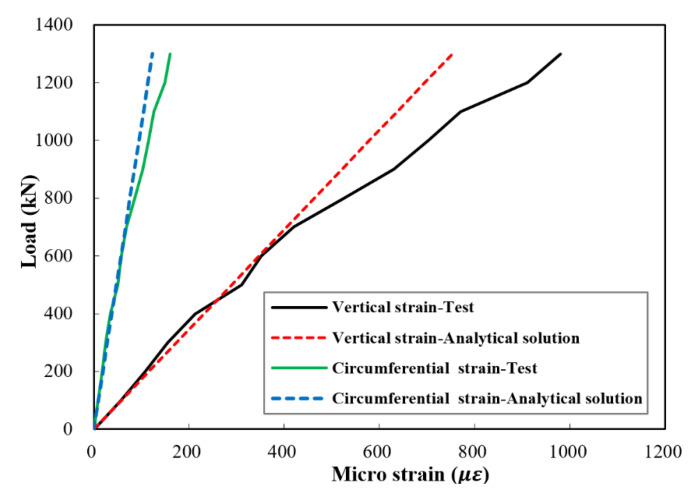
Comparison between test results and analytical solution of the vertical strain and the circumferential strain of UHPC jacket of specimen J2.

The vertical strain of the stub column and the circumferential strain of the UHPC jacket can be computed by Equations (30) and (37), respectively. A comparison of the analytical and experimental values of the elastic vertical and circumferential strains is given in Figure 15 and Figure 16. It can be noticed that the difference in the circumferential strains was smaller than that in the vertical strains. Additionally, the values obtained in specimen J1 were closer to the analytical solution than in J2. In general, compared with the experimental results, the analytical solution gave close results with an average difference of 4% and a maximum difference of about 12%.

Another coefficient, *β*, is introduced as the circumferential strain coefficient of the UHPC jacket. Here, *β* is defined as the ratio of the vertical strain of the concrete stub column *ε*_*z*1_ to the circumferential strain of the UHPC jacket *ε*_*θ*1_:

(43) $$\beta =\frac{\varepsilon_{z1}}{\varepsilon_{\theta 2}}$$

The meaning of *β* is that whenever there is a vertical strain *ε*_*z*1_ in the concrete stub column, a simultaneous corresponding circumferential strain *ε_θ_*_2_ in the UHPC jacket follows. Therefore, the circumferential strain in the UHPC jacket *ε_θ_*_2_ can be easily determined if the value of *β* is known. The coefficient *β* can be calculated from the material properties and geometry of the column. 

By substituting Equations (30) and (37) into Equation (43), the following is obtained:

(44) $$\beta =\frac{\varepsilon_{z1}}{\varepsilon_{\theta 2}}=-\frac{\lambda +\mu_{2}+\left(1+\mu_{1}\right)\left(1-2\mu_{1}\right)n_{E}}{\mu_{1}(\lambda +\mu_{2})}$$

**Figure 17:**
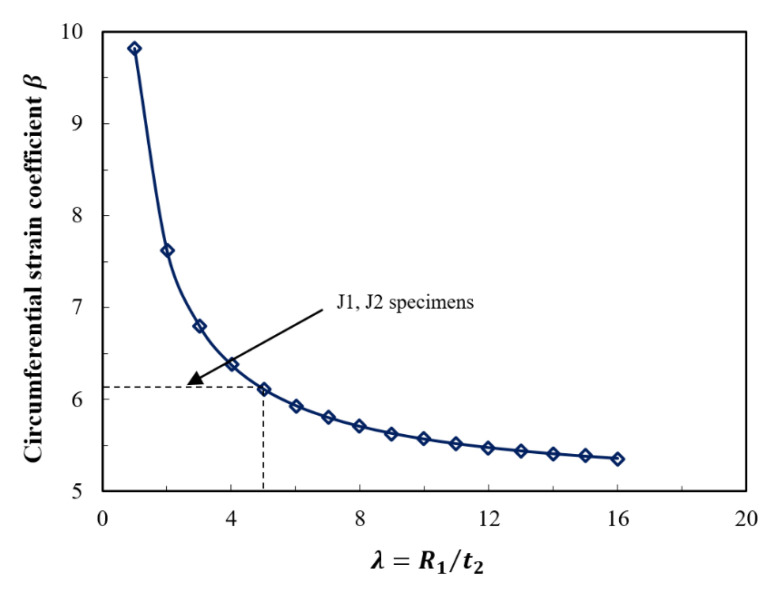
Effect of column radius to jacket thickness ratio on the circumferential strain coefficient *β*.

The influence of the circumferential strain coefficient *β* with regard to a variable column radius to jacket thickness ratio *λ* is shown in Figure 17. The relationship had a reverse exponential trend in which the circumferential strain coefficient *β* increased as the value of *λ* became smaller. In other words, the increase in the confinement was proportional to the increase in the jacket thickness. When λ had a value greater than 5, the change in *β* became very slight, indicating that jacketing with a very small thickness became insignificant. Hence, a minimum limit for the thickness could be determined based on *β*. For the tested specimens, J1 and J2, the mean value of the coefficient *β* was 6.1.

## 5. Conclusions

In this study, an analytical solution based on the theory of elasticity was proposed and expressions for calculating the stresses and strains of concrete circular columns with UHPC jackets were derived. In addition, three small-scale columns, two of which with UHPC jackets, were tested under axial compression. Discussion of the influence of UHPC jacketing was provided, and verification of the proposed solution for estimating the elastic response was demonstrated. From the analytical and experimental investigations, the following conclusions were drawn:

- UHPC jacketing was proven to be a viable retrofitting method that not only improves or restores the strength of concrete circular columns but also efficiently enhances the elastic behaviour, which is paramount when members are designed to maintain an elastic state under the increase in service loads.
- The increase in the elastic enhancement was reflected by the restraint effect coefficient *K*, which increased with the increase in the UHPC jacket thickness. Based on the experimental data in this study, a value of 14% was achieved when a column diameter to jacket thickness ratio *λ* = 6 was used.
- UHPC jacketing was shown to be more significant in the nonlinear stage, where the load-bearing capacity increased by 50%, and the failure patterns tended to be gradually slow rather than abruptly brittle. Besides, the effectiveness of the UHPC jacket in the elastic stage was noticed to have a non-negligible improvement.
- Compared with the measured test data, the analytical solution derived herein gave good predictions of the stresses and strains in the elastic stage, and the value of *β* could be used as a theoretical basis for the design of the jacket.

This study was an attempt to explore the potentials of using UHPC as a jacketing technique for concrete columns. The work here predominantly concentrated on the elastic performance of circular columns with UHPC jackets. This work serves as a clear and straightforward theoretical and experimental reference for computing the elastic confinement effect provided by jackets, and its application could be extended to materials other than UHPC. Further parametric and large-scale experimental research is still needed for investigating the effectiveness and limitations of UHPC jackets in retrofitting columns.

## Figures and Tables

**Table 1:** UHPC mixture proportions for one cubic meter.

Constituent	Proportion (kg)
Powder	2095
Steel fibres	156
Superplasticizer	22.1
Water	182.4

**Table 2:** Material properties for normal concrete.

Property	Specimen Geometry	Test Results
Cubic compressive strength (MPa)	150 × 150 × 150	44.2
Modulus of Elasticity (MPa)	–	34,500
Tensile strength (MPa)	–	2.79
Passion’s ratio	–	0.167

**Table 3:** Material properties for UHPC.

Property	Specimen Geometry	Test Results
Cubic compressive strength (MPa)	100 × 100 × 100	156.8 (MPa)
Modulus of Elasticity (MPa)	100 × 100 × 300	55,468 (MPa)
Flexural strength (MPa)	100 × 100 × 400	17.2 (MPa)
Poisson’s ratio ^a^	–	0.2

Note: ^a^ the value is adopted from AFGC [56].

**Table 4:** Summary of test results.

Specimen	Ultimate Load (kN)	Ultimate Compressive Strength (MPa)	Ultimate Displacement (mm)	Ultimate Axial Strain (με)
RC	1850	36.69	1.219	2031
J1	2800	57.07	2.311	3852
J2	2650	54.01	2.227	3712

## Data Availability

All data, models, or codes generated or used during the study appear in the published article.

## References

[1] Shi X., Xie N., Fortune K., Gong J. (2012). Durability of Steel Reinforced Concrete in Chloride Environments: An Overview. Constr. Build. Mater..

[2] Li Q., Wang C. (2015). Updating the Assessment of Resistance and Reliability of Existing Aging Bridges with Prior Service Loads. J. Struct. Eng..

[3] Horszczaruk E. (2005). Abrasion Resistance of High-Strength Concrete in Hydraulic Structures. Wear.

[4] Raza S., Khan M.K., Menegon S.J., Tsang H.-H., Wilson J.L. (2019). Strengthening and Repair of Reinforced Concrete Columns by Jacketing: State-of-the-Art Review. Sustainability.

[5] Ong K.C.G., Kog Y.C., Yu C.H., Sreekanth A.P. (2004). V Jacketing of Reinforced Concrete Columns Subjected to Axial Load. Mag. Concr. Res..

[6] Mohamed Sayed A., Mohamed Rashwan M., Emad Helmy M. (2020). Experimental Behavior of Cracked Reinforced Concrete Columns Strengthened with Reinforced Concrete Jacketing. Materials.

[7] Anand P., Sinha A.K. (2020). Effect of Reinforced Concrete Jacketing on Axial Load Capacity of Reinforced Concrete Column. Civ. Eng. J..

[8] Hollaway L.C. (2010). A Review of the Present and Future Utilisation of FRP Composites in the Civil Infrastructure with Reference to Their Important In-Service Properties. Constr. Build. Mater..

[9] Mancusi G., Feo L., Berardi V.P. (2012). Concrete Open-Wall Systems Wrapped with FRP under Torsional Loads. Materials.

[10] Pham T.M., Hadi M.N., Youssef J. (2015). Optimized FRP Wrapping Schemes for Circular Concrete Columns under Axial Compression. J. Compos. Constr..

[11] Matthys S., Toutanji H., Taerwe L. (2006). Stress–Strain Behavior of Large-Scale Circular Columns Confined with FRP Composites. J. Struct. Eng..

[12] Mirmiran A., Shahawy M. (1997). Behavior of Concrete Columns Confined by Fiber Composites. J. Struct. Eng..

[13] George J., Sreekala M.S., Thomas S. (2001). A Review on Interface Modification and Characterization of Natural Fiber Reinforced Plastic Composites. Polym. Eng. Sci..

[14] Toutanji H., Ortiz G. (2001). The Effect of Surface Preparation on the Bond Interface between FRP Sheets and Concrete Members. Compos. Struct..

[15] Cabral-Fonseca S., Correia J.R., Custódio J., Silva H.M., Machado A.M., Sousa J. (2018). Durability of FRP-Concrete Bonded Joints in Structural Rehabilitation: A Review. Int. J. Adhes. Adhes..

[16] Zhang P., Hu Y., Pang Y., Gao D., Xu Q., Zhang S., Sheikh S.A. (2020). Experimental Study on the Interfacial Bond Behavior of FRP Plate-High-Strength Concrete under Seawater Immersion. Constr. Build. Mater..

[17] Wu Y.-F., Liu T., Oehlers D.J. (2006). Fundamental Principles That Govern Retrofitting of Reinforced Concrete Columns by Steel and FRP Jacketing. Adv. Struct. Eng..

[18] Choi E., Chung Y.-S., Park J., Cho B.-S. (2010). Behavior of Reinforced Concrete Columns Confined by New Steel-Jacketing Method. ACI Struct. J..

[19] Ghobarah A., Biddah A., Mahgoub M. (1997). Rehabilitation of Reinforced Concrete Columns Using Corrugated Steel Jacketing. J. Earthq. Eng..

[20] Guedes Soares C., Garbatov Y., Zayed A. (2011). Effect of Environmental Factors on Steel Plate Corrosion under Marine Immersion Conditions. Corros. Eng. Sci. Technol..

[21] Bing W., Xiaoping C., Shujun J., Qingyou L. (2015). Steel Plate with Corrosion Resistance to High Humid and Hot Marine Atmosphere and Manufacturing Method Thereof.

[22] Trapko T. (2013). Fibre Reinforced Cementitious Matrix Confined Concrete Elements. Mater. Des..

[23] Ilki A., Demir C., Bedirhanoglu I., Kumbasar N. (2009). Seismic Retrofit of Brittle and Low Strength RC Columns Using Fiber Reinforced Polymer and Cementitious Composites. Adv. Struct. Eng..

[24] Rubino F., Nisticò A., Tucci F., Carlone P. (2020). Marine Application of Fiber Reinforced Composites: A Review. J. Mar. Sci. Eng..

[25] Lee K.S., Lee B.Y., Seo S.Y. (2016). A Seismic Strengthening Technique for Reinforced Concrete Columns Using Sprayed FRP. Polymers.

[26] Ates A.O., Khoshkholghi S., Tore E., Marasli M., Ilki A. (2019). Sprayed Glass Fiber–Reinforced Mortar with or without Basalt Textile Reinforcement for Jacketing of Low-Strength Concrete Prisms. J. Compos. Constr..

[27] Gong T., Heravi A., Alsous G., Curosu I., Mechtcherine V. (2019). The Impact-Tensile Behavior of Cementitious Composites Reinforced with Carbon Textile and Short Polymer Fibers. Appl. Sci..

[28] Al-Gemeel A.N., Zhuge Y. (2019). Using Textile Reinforced Engineered Cementitious Composite for Concrete Columns Confinement. Compos. Struct..

[29] Shang X., Yu J., Li L., Lu Z. (2019). Strengthening of RC Structures by Using Engineered Cementitious Composites: A Review. Sustainability.

[30] Li V.C., Horii H., Kabele P., Kanda T., Lim Y. (2000). Repair and Retrofit with Engineered Cementitious Composites. Eng. Fract. Mech..

[31] Reggia A., Morbi A., Plizzari G.A. (2020). Experimental Study of a Reinforced Concrete Bridge Pier Strengthened with HPFRC Jacketing. Eng. Struct..

[32] Cho C.-G., Han B.-C., Lim S.-C., Morii N., Kim J.-W. (2018). Strengthening of Reinforced Concrete Columns by High-Performance Fiber-Reinforced Cementitious Composite (HPFRC) Sprayed Mortar with Strengthening Bars. Compos. Struct..

[33] Mateckova P., Bilek V., Sucharda O. (2021). Comparative Study of High-Performance Concrete Characteristics and Loading Test of Pretensioned Experimental Beams. Crystals.

[34] Shann S.V. (2012). Application of Ultra High Performance Concrete (UHPC) as a Thin-Bonded Overlay for Concrete Bridge Decks. Master’s Thesis.

[35] Zhou M., Lu W., Song J., Lee G.C. (2018). Application of Ultra-High Performance Concrete in Bridge Engineering. Constr. Build. Mater..

[36] Li J., Wu C., Hao H., Liu Z. (2017). Post-Blast Capacity of Ultra-High Performance Concrete Columns. Eng. Struct..

[37] Abbas S., Nehdi M.L. (2021). Mechanical Behavior of Ultrahigh-Performance Concrete Tunnel Lining Segments. Materials.

[38] Graybeal B.A. (2005). Characterization of the Behavior of Ultra-High Performance Concrete. Ph.D. Thesis.

[39] Haber Z.B., De la Varga I., Graybeal B.A., Nakashoji B., El-Helou R. (2018). Properties and Behavior of UHPC-Class Materials.

[40] Marani A., Jamali A., Nehdi M.L. (2020). Predicting Ultra-High-Performance Concrete Compressive Strength Using Tabular Generative Adversarial Networks. Materials.

[41] Ichikawa S., Matsuzaki H., Moustafa A., ElGawady M.A., Kawashima K. (2016). Seismic-Resistant Bridge Columns with Ultrahigh-Performance Concrete Segments. J. Bridge Eng..

[42] Tong T., Wang J., Lei H., Liu Z. (2020). UHPC Jacket Retrofitting of Reinforced Concrete Bridge Piers with Low Flexural Reinforcement Ratios: Experimental Investigation and Three-Dimensional Finite Element Modeling. Struct. Infrastruct. Eng..

[43] Tong T., Lei H., Yuan S., Liu Z. (2020). Experimental Investigation and Seismic Vulnerability Assessment of Low Flexural Strength Rectangular Bridge Piers Retrofitted with Ultrahigh-Performance Concrete Jackets. Eng. Struct..

[44] Aaleti S., Sritharan S., Abu-Hawash A., Toutlemonde F., Resplendino J. (2013). Innovative UHPC-Normal Concrete Composite Bridge Deck. Proceedings of the RILEM-fib-AFGC International Symposium on Ultra-High Performance Reinforced Concrete.

[45] Graybeal B.A., Tanesi J. (2007). Durability of an Ultrahigh-Performance Concrete. J. Mater. Civ. Eng..

[46] Peter R. Cost-Effectiveness and Sustainability of UHPC. Proceedings of the International Symposium on Ultra High Performance Concrete.

[47] Sheheryar M., Rehan R., Nehdi M.L. (2021). Estimating CO_2_ Emission Savings from Ultrahigh Performance Concrete: A System Dynamics Approach. Materials.

[48] Sedran T., Durand C., Larrard F.D. An Example of UHPFRC Recycling. Proceedings of the International Conference on Designing and Building with UHPFRC, State of the Art and Development.

[49] Doiron G. (2016). Pier Repair/Retrofit Using UHPC—Examples of Completed Projects in North America. International Interactive Symposium on Ultra-High Performance Concrete.

[50] Farzad M., Shafieifar M., Azizinamini A. (2019). Retrofitting of Bridge Columns Using UHPC. J. Bridge Eng..

[51] Farzad M., Sadeghnejad A., Rastkar S., Moshkforoush A., Azizinamini A. (2020). A Theoretical Analysis of Mechanical and Durability Enhancement of Circular Reinforced Concrete Columns Repaired with UHPC. Eng. Struct..

[52] Xie J., Fu Q., Yan J.-B. (2019). Compressive Behaviour of Stub Concrete Column Strengthened with Ultra-High Performance Concrete Jacket. Constr. Build. Mater..

[53] Dadvar S.A., Mostofinejad D., Bahmani H. (2020). Strengthening of RC Columns by Ultra-High Performance Fiber Reinforced Concrete (UHPFRC) Jacketing. Constr. Build. Mater..

[54] Timoshenko S., Goodier J.N. (1951). Theory of Elasticity.

[55] GB/T 50152-2012 (2012). Standard for Test Method of Concrete Structures.

[56] Association Française de Génie Civil (AFGC)/Service d’études techniques des routes et autoroutes (SETRA) (2002). Bétons Fibrés à Ultra-Hautes Performances, Recommandations Provisoires.

[57] Attiogbe E.K., Darwin D. (1987). Submicrocracking in Cement Paste and Mortar. Mater. J..

